# Using fertile couples as embryo donors: An ethical dilemma

**Published:** 2014-03

**Authors:** Leila Alizadeh, Reza Omani Samani

**Affiliations:** 1 *Medical Ethics and Law Research Center, Shahid Beheshti University of Medical Sciences, Tehran, Iran.*; 2 *Department of Epidemiology and Reproductive Health at Reproductive Epidemiology Research Center, Royan Institute for Reproductive Biomedicine, ACECR, Tehran, Iran.*; 3 *Medical Ethics and History of Medicine Research Center, Tehran University of Medical Sciences, Tehran, Iran.*

**Keywords:** *Embryo*, *Gamete*, *Donation*, *Legal*, *Ethical*, *Religious*, *Frozen embryo*, *Fertile couples*

## Abstract

The use of donated embryos has offered hope for infertile couples who have no other means to have children. In Iran, fertility centers use fertile couples as embryo donors. In this paper, the advantages and disadvantages of this procedure will be discussed. We conclude that embryo-donation should be performed with frozen embryos thus preventing healthy donors from being harmed by fertility drugs. There must be guidelines for choosing the appropriate donor families. In countries where commercial egg donation is acceptable, fertile couples can be procured as embryo donors thus fulfilling the possible shortage of good quality embryos. Using frozen embryos seems to have less ethical, religious and legal problems when compared to the use of fertile embryo donors.

## Introduction

The use of donated embryos for the purpose of establishing a family is a successful therapeutic option for some infertile couples. As with gamete donation, embryo donation has resulted in the birth of many children over the 27 years that the procedure has been used. Comparing to the gamete donation, use of donated frozen embryos is less complex and less expensive. It can also provide the donors a sense of altruism as their act helps other patients establish complete family with children although a family necessarily does not include children. Embryo cryopreservation is an unavoidable part of assisted reproduction technology (ART) which normally is for future usage, so the number of embryos in storage is increasing throughout the world (1-3). 

These good quality, surplus embryos are usually stored for subsequent treatment cycles and normally utilized by the owner couples, thus resulting in a significant increase in the cumulative pregnancy rate per oocyte collection (4, 5). Some couples choose not to use a portion or all of their cryopreserved embryos, of which the most common reason is the completeness of their families (4, 6). Therefore, a number of the embryos are not used until maximum storage limit (4). Published reports about the numbers of frozen embryos in storage have ranged from 52000 (UK, 1996), 71000 (Australia, 2000) and 400000 (USA, 2003) (7). 

It is reported that the duration of cryostorage does not affect post-thaw embryos either on their survival or on pregnancy outcome; hence, the length of time embryos could remain in storage is subject to legislation which varies in different countries (8-15). In Iran, legislation regarding the length of the cryopreservation period does not exist. Therefore, in this regard decisions regarding the duration of conserving surplus embryos are made by the ART centers. 

The Iranian Parliament passed the embryo donation law which was followed by approval from the Iranian Guardian Council in 2003. In 2005, the Ministry of Health established the legislation for ART centers regarding this procedure. According to this law, infertility centers can donate embryos from any legal married couple to the infertile ones once permission is obtained from the donor couples. Currently in Iran, centers obtain embryos from normal fertile couples for embryo donation (16). There seems to be many ethical, religious and legal problems regarding embryo donation from fertile couples as donors like putting normal couples in risk, anonymity, payment and repetitive donation. Our focus in this paper is on recruiting normal and fertile couples, put them in the ART procedure and obtain their gametes for making embryos and donating the resulting embryos to other infertile couples.

**Definitions and history**

The first successful oocyte donation was reported in 1984 at about the same time as the first embryo donation (17, 18). Sperm donation however, began a number of years ago (19). Embryo donation can be defined as the transfer of an embryo resulting from spermatozoa and oocytes fertilization which is not from the recipient and her partner (1, 20). Most of the time, it refers to the donation of surplus frozen embryos from an infertile couple to another couple. If fresh embryos from “healthy fertile couples” who volunteer for donation are used instead of surplus frozen embryos, this could be equal to “synchronous spermatozoa and oocyte donation”. This procedure is sometimes referred to “both gamete donation” (21). It means getting oocytes from the wife and spermatozoa from the husband make embryos from them and transfer the resulting embryos to another infertile couple. It is forbidden in some countries, such as France but there is nothing against it in Iranian law (3).

**Iran’s law about embryo donation and guideline**

The bill of embryo donation was ratified in the Iranian Parliament and Guardian Council on 07/20/2003. It has five articles and focuses on the recipients’ social and health situation. The law talks less about the donor except they must be legally and religiously married couples (22). The executive guideline of the mentioned law was communicated to the ART centers on 03/15/2005. In this guideline more details are presented about the donors’ social and health situation. Some criteria like good health, acceptable IQ, no addiction and no incurable disease are mentioned as donors’ criteria. 

Religious conformity between donor and recipient is also mentioned (23). Also no compensation is accepted for the embryo donors in this guideline. Now the question is: “Is there any concern about using fertile couples as embryo donors in Iran’s law?” The answer is no. The only concern of Iran’s embryo donation law is legality and religious acceptability of the donor’s marriage. After an inquiry from Iran’s Guardian Council about whether “this law includes fertile couples as donors or not?” the answer was: “all the legally and religiously married couples are eligible to donate their embryos”. It means that centers can use fertile couples as embryo donors. 

**Indications of embryo donation in Iran**

It is well known that the embryo donation is indicated when there is no possibility of pregnancy in the absence of this technique, when other treatments have failed or have a minimal chance of success, or if there is a risk of transmission of a serious genetic disease if pre-implantation genetic diagnosis is neither feasible nor acceptable (24). The most frequent indication could be premature or incipient ovarian failure in combination with severe male factor infertility, in other words, a “lack of gametes from both partners” (25). 

On the contrary, in Iran due to some cultural and religious concerns, sperm donation is replaced by embryo donation. Although there are decrees from the clergy leaders permitting the sperm donation and considering that the sperm in embryo donation is as strange as in sperm donation, our infertile men do not accept this procedure. It seems strange but, Iranian men don’t have good feeling about using sperm of another man, but can accept the embryo donation because it is called “embryo” and not called “sperm” (26). 

As a result, it is reported that more than 90% of embryo requests are from couples with severe male factor infertility (27, 28). This condition increases the demand for embryos as almost all the couples with the indication for sperm donation are included with embryo donation candidates. The fact is that infertility centers are performing embryo donations from fertile couples to fulfill the increased demand. 

**Disadvantages of using fertile couples as embryo donors**

**Medical risk**

Induction of ovulation is necessary for the donor and these medications have a variety of complications which range from the risk of cancer to potential life threatening situations such as hyper-stimulation syndrome which, in turn, may cause complications like deep vein thrombosis (29-35). The practice of egg donation in Iran is practiced, and its complications are unavoidable (36). However the risks in egg donation and embryo donation for women are the same. With the mentioned explanation, embryo donation from fertile couples can be considered acceptable, however when we have abundant numbers of surplus embryos, it seems unethical to use fertile couples and place the women at risk solely for their embryos.

**Payment**

As previously discussed, Iranian law does not permit any payment for embryo donation. On the contrary, payments to the egg donors are completely acceptable and practiced in Iran. Considering the donation procedure, there is no difference between egg donors and embryo donor women, because they follow the same procedure and undergo the same treatment and take the same risks. Morality of commercial donation is not the issue of this paper, however, as we know, even in some countries that do not accept commercial donation such as the UK, compensation for the donor’s time, participation in lab tests and procedures, suffering from surgery and anesthesia can be acceptable (37). 

The point is, when fertile couples are chosen for embryo donation, in actuality we hire an egg and sperm donor with full process of the donation for both (21). Thus, in the presence of compensation for egg donors, it would be unacceptable not to pay the fertile couples for their donation, in particular the wife, who is the same as an egg donor. Therefore, the choice is either unethically not to compensate the donors and follow the law, or ethically compensate the donors and break the law. Also, it is necessary to state that without payment, donors will definitely lose their motivation thus causing a shortage of embryo donors. 

On the other hand, when frozen embryos are used for donation, donors are not compensated, because all the expenses for ART including drugs, procedures, time and etc. which they paid, were for their own treatment, not just for donation and after the cryopreservation time, the surplus embryos has no use for them. Moreover the altruistic incentives can be increased in the frozen embryo donation, as the owners of the embryos do not need them anymore and donate them just for saving other infertile families and give the embryos the chance to live (11, 38). However, when considering fee to be paid to the donors, we shall expect the following consequences.

A) Advertisement: Advertisers and brokers are an important issue in commercial donation, something which contrasts human dignity. Financial benefit usually is the main reason for advertisement, which in turn can encourage many healthy fertile couples to participate in donation programs (39). Using surplus frozen embryos based on legal principles does not have any financial benefit and is used only for altruistic aims; hence brokers and advertisers are eliminated.

B) Monetary contract, monetary litigation: The lack of enforceability of contracts, absence of full law protection, lack of commitment for providing full information for the donors and recipients may cause difficulties in donation programs. In these circumstances, many arguments could happen resulting in the creation of many court cases. Due to the lack of an appropriate law, the courts cannot properly judge in order to protect families. None of the above mentioned issues happens if surplus frozen embryos are used without payment.

C) Repetitive oocyte donation: It has been reported that repetitive donation has adverse effects on the oocyte donor and should be limited to less than six donations. Without legal limitation, people may want to donate more for the purpose of making money, thus putting themselves in danger. Also, the large number of donations results in larger numbers of siblings which has its own consequences (40). This issue is very important from Islamic perspective also. The use of frozen embryos does not encourage fertile people to donate repetitively. 

**Religious issues**

The religious review is done from Islamic texts and decrees as the authors could gather. The most important note is the egg donation and embryo donation has similar Islamic controversies that are not discussed here; our focus is on the differences between embryo donation from fertile donors and frozen embryo donation. Donation is legal and religiously accepted in Iran. Many of our clergy leaders have issued the decree confirming egg and embryo donation but in the condition that no other forbidden act is done for donation. In Islam, looking at and touching the genitalia is forbidden except for the spouse, but when there is a necessity for treatment, this is permitted for doctors (21). 

Here, we should discuss what necessity is and which conditions can be referred to as diseases. Then, infertility can be discussed in terms of a disease or an enhancement. Clergy leaders believe that this necessity is not just a request, but a hard situation in which life is difficult for the patient. Islamic clergies believe that infertility is a disease and should be treated, thus the first decree “Fatwa” that accepted the use of ART for infertile couples was released in 1980, just two years after the birth of the first IVF baby (41-45). In our country and most countries in and around the Middle East, infertility is a difficult situation leading to serious family problems or divorce (46).

So, it is clear that infertility is a disease to be treated and can be referred to as a necessity that permits the doctor to look at and touch the genitalia as a part of infertility treatment. Donation programs are practiced in Iran but no other Islamic country allows this kind of treatment. Look and touch can be accepted for donation even if a necessity does not exist for the donor, because the donor is healthy and solely donating for treatment of the other person. Our clergies believe that as with the case of blood donation, the necessity for treatment of an infertile couple that has no other way except for donation, makes necessity for the donors who are sacrificing to save a family. So look and touch can be acceptable for the donor. Hence, we could assume that look and touch in embryo donation from healthy fertile couples can be acceptable, but only when there is no alternative for obtaining embryos. Considering the nitrogen tanks full of frozen embryos, it seems that the necessity for treatment of fertile donors is questionable. 

**Potential advantages of embryo donation from fertile couples**

**Fresh or frozen embryo**

There is a tendency to choose a fresh embryo for obtaining a higher success rate, but with the improvement in embryo cryopreservation techniques, it seems that cumulative probability of pregnancy following fresh or frozen embryo transfer is similar (47-49). Also embryo freezing does not adversely affect prenatal outcome in terms of prematurity, low birth weight and small for gestational age versus fresh embryo transfer. The outcome is similar or even better, particularly regarding fetal growth (50). Although the children born from frozen embryos are still young and more time is needed to fully confirm the procedure, it seems that fresh embryo transfer is not so advantageous that it encourages embryo transfer from fertile couples. There is also a term “embryo-sharing” that was defined by Samani *et al* (21). In this program infertile couples donate one of their fresh embryos to another infertile couple with altruistic incentives. 

**Choice and screening**

The major concern with gamete and embryo donation is donor screening. It is true that the majority of frozen embryo donors are infertile. Although the screening procedure is possible for fertile donors due to their availability, however it is not easy to locate couples who have stored their embryos for future evaluation. Moreover, we cannot consider that infertile couples are completely healthy and may have some known genetic or chromosomal diseases (3, 51). During the past decades, several reports have declared that the risk of gene mutations may be increased in assisted reproduction offspring, even though their fathers are of normal spermatogenesis and genetic backgrounds (52). The etiology of compromised spermatogenesis is often genetic (53). 

Genetic origins also play a role in many other infertility causes, such as polycystic ovaries (54). In addition, the mean-age of infertile couples who are undergoing ARTs is significantly higher than potential fertile donors, which raises a concern about possible abnormalities (55). Normally, infertile patients are evaluated for their own treatment which is perhaps less than donors and the health of embryos even from fertile people cannot be guaranteed. However, we can choose donors of younger ages and more evaluated patients. It would be more favorable if we include donors from patients with positive results from their IVF treatments in the absence of detectable abnormalities in their children. 

**Principles of medical ethics**

Four principles of medical ethics presented by Beauchamp and Childress are well known by the physicians, but sometimes in the practice they conflict with each other and a dilemma appears (56). Here in our case, respect for autonomy means the freedom of the recipients to choose the fresh embryos from fertile donors rather than surplus frozen embryos from infertile patients. Also, justice in this situation probably means that a same chance must be provided for every embryo recipient to reach to an embryo and have a child. Considering limited source of the frozen embryos, it seems that without hiring fertile donors justice is not provided for the recipients and if their autonomy is respected, whenever they choose fertile donors, it should be performed. These two principles are in conflict with beneficence and non-maleficence principles if we look at the donors. 

Actually we have two patient couples here, recipients and fertile donors that we must consider the ethical principles for both. The main argument here is "the necessity" that we can do ethical judgment upon it. The necessity here is the "need of the recipients" which makes the doctor to accept putting the donor in danger. If the frozen embryo can be provided, with new reports stating that frozen and fresh embryos has same chance for pregnancy there is no need to put someone in danger for "respect for autonomy" and "justice" is already looked after (47-50). But if the number of frozen embryos is not adequate for the recipients, and egg donation is accepted, hiring fertile donors seems to be ethical if the above mentioned unethical consequences are solved by proper law and legislations.

## Conclusion

It seems that in countries where egg donation and donor compensation is accepted, embryo donation from fertile donors may also be acceptable. It is important to note that placing fertile people at medical risk can only be acceptable in the absence of another alternative. Thus, when there are numerous frozen embryos ready for donation it is not justifiable to place people at risk just for embryo donation. More detailed legislation should be created and added to the current embryo donation law with the purpose of protecting infertile families and alleviating their problems.
